# Predicting prognosis of nasopharyngeal carcinoma based on deep learning: peritumoral region should be valued

**DOI:** 10.1186/s40644-023-00530-5

**Published:** 2023-02-09

**Authors:** Song Li, Xia Wan, Yu-Qin Deng, Hong-Li Hua, Sheng-Lan Li, Xi-Xiang Chen, Man-Li Zeng, Yunfei Zha, Ze-Zhang Tao

**Affiliations:** 1 Department of Otorhinolaryngology, The First Affiliated Hospital, Nanjing Medical University, Nanjing, 210029 China; 2 Department of Otolaryngology-Head and Neck Surgery, Renmin Hospital of Wuhan University, 238 Jie-Fang Road, Wuhan, Hubei 430060 P.R. China; 3 Department of Otolaryngology-Head & Neck Surgery, Ezhou Central Hospital, No. 9 Wenxing Road, Ezhou, 436000 P.R. China; 4 Department of Radiology, Renmin Hospital of Wuhan University, 238 Jie-Fang Road, Wuhan, Hubei 430060 P.R. China

**Keywords:** Peritumoral region, Deep learning, Nasopharyngeal carcinoma, Prognosis prediction

## Abstract

**Background:**

The purpose of this study was to explore whether incorporating the peritumoral region to train deep neural networks could improve the performance of the models for predicting the prognosis of NPC.

**Methods:**

A total of 381 NPC patients who were divided into high- and low-risk groups according to progression-free survival were retrospectively included. Deeplab v3 and U-Net were trained to build segmentation models for the automatic segmentation of the tumor and suspicious lymph nodes. Five datasets were constructed by expanding 5, 10, 20, 40, and 60 pixels outward from the edge of the automatically segmented region. Inception-Resnet-V2, ECA-ResNet50t, EfficientNet-B3, and EfficientNet-B0 were trained with the original, segmented, and the five new constructed datasets to establish the classification models. The receiver operating characteristic curve was used to evaluate the performance of each model.

**Results:**

The Dice coefficients of Deeplab v3 and U-Net were 0.741(95%CI:0.722–0.760) and 0.737(95%CI:0.720–0.754), respectively. The average areas under the curve (aAUCs) of deep learning models for classification trained with the original and segmented images and with images expanded by 5, 10, 20, 40, and 60 pixels were 0.717 ± 0.043, 0.739 ± 0.016, 0.760 ± 0.010, 0.768 ± 0.018, 0.802 ± 0.013, 0.782 ± 0.039, and 0.753 ± 0.014, respectively. The models trained with the images expanded by 20 pixels obtained the best performance.

**Conclusions:**

The peritumoral region NPC contains information related to prognosis, and the incorporation of this region could improve the performance of deep learning models for prognosis prediction.

## Background

Tremendous advances in computer vision technology in recent years have enabled artificial intelligence (AI) to extract valuable feature information from medical images with increasing efficiency, which has led to an accelerated integration of AI into the medical field [1]. Convolutional neural network (CNN), owing to its remarkable image feature extraction capability, is one of the most used AI techniques in medical imaging. Research on the application of AI based on CNN technology in the field of nasopharyngeal carcinoma, including image segmentation [2], image classification and recognition [3], drug efficacy prediction [4, 5], and prognosis prediction [6, 7], has also gradually increased in recent years and has generally shown better performance than traditional machine learning methods. Many studies have reported its prediction performance in nasopharyngeal carcinoma (NPC) prognosis to exceed the traditional TNM staging system [8, 9].

Training artificial intelligence (AI) models to predict the prognosis of nasopharyngeal carcinoma (NPC) has been a focus of research in recent years [10]. Radiomics [11–14] and deep learning (DL) [15–17] are the main methods used in most studies, despite the use of deep learning-based radiomics [4, 18]. The main advantage of radiomics is that the model is well interpretable and requires less data, and the main defect is that the segmented area must be outlined manually, which is labor-consuming [19]. Considering that one of the biggest challenges in oncology is the development of accurate and cost-effective screening procedures [20], this labor-intensive step hinders its clinical utility [21]. DL method enable fully automated analysis of images; however, a large number of well-labeled images are required. Predicting tumor prognosis requires patient endpoint information, which is costly and time consuming to collect; this leads to high data costs for building DL models. Therefore, training better-performing models based on limited datasets is currently an important problem to be solved in the AI community [22, 23]. Building models that can extract valuable features from medical images more efficiently using medical priori knowledge as a guide is one of the solutions.

The TNM staging system is used clinically to evaluate the prognosis of NPC. In this system, the T-staging is focused on the information of the relationship between the tumor and the surrounding anatomical structures [24, 25]. Many studies have indicated that T-staging achieves considerable performance in predicting tumor prognosis [9, 26], which confirms the predictive value of the peritumoral region. However, the prediction models established by radiomics are conventionally based on the tumor region, in which the peritumoral region is erased. In contrast, the predictive models built by DL are mainly based on full images containing distant information that is generally considered clinically irrelevant to tumor prognosis [25]. We believe that the peritumoral region, not only the tumor region, should receive additional attention when applying AI to predict the prognosis of NPC. Therefore, we envisioned that incorporating the tumor region along with a portion of the peritumoral region for analysis may contribute to improvement of the performance of the predictive DL model. This study was designed to validate this idea.

## Methods

### Patients and images

Patients with pathology-confirmed NPCs who were admitted for treatment between June 2012 and December 2018 were retrospectively selected. Patients were screened according to the inclusion and exclusion criteria presented in Table 1. Pre-treatment magnetic resonance imaging (MRI) images, including axial T1-enhanced sequences, were collected from the picture archiving and communication system for all included patients, and demographic information was tabulated simultaneously. Progression-free survival (PFS), defined as the time from treatment to disease progression (recurrence or distant metastasis) or the occurrence of death caused by any reason or the last review, was regarded as the endpoint. The standard for regular review was defined as follows: Patients were reviewed at least every 3 months for 2 years after treatment, at least every 6 months from 2 to 5 years after treatment, and annually starting 5 years after treatment. Items to be included in the review were MRI of nasopharyngeal, CT/MRI of the head and abdomen or contingent positron emission tomography (PET)/CT. Patients who stopped reviewing after 2 years of regular review were followed up by telephone. Recurrence was confirmed on the basis of nasopharyngeal MRI or pathology obtained via endoscopy. Criteria for determining recurrence based on MRI of the nasopharynx were defined as progressive localized bone erosion being reported, areas of abnormal soft tissue larger than those reported on the previous review, and intensive shadows newly identified in the previous neck review that were progressively increasing in the current review. The results of lung CT, CT/MRI of the head and abdomen, or PET/CT were used to determine the presence of distant metastases. Fatality data were obtained by telephone follow-up. MRI was obtained using 3.0-T MR imaging systems (GE, Discovery MR 750 and Signa HDxt). Axial T1-enhanced sequence was used to construct the datasets. The parameters for the images were as follows: repetition time 552–998 msec, echo time 9.65–13.79 msec, flip angle 90-111°, slice thickness 4–7 mm, and matrix size 512*512. This study was approved by the Ethics Committee of author’s hospital, and informed consent from patients was waived.

**Table 1:** Inclusion and exclusion criteria of patients

Inclusion criteria	• Primary nasopharyngeal carcinoma diagnosed by pathology and treated in hospital. • No distant metastases at the time of initial diagnosis • Pre-treatment MRI images were available, which included axial T1-enhanced sequences • Regular review was performed • Telephone follow-up was available
Exclusion criteria	• nasopharyngeal carcinoma that recurred after treatment • Pre-treatment MRI was not available, or the images were corrupted, or ther have not axial T1-enhanced sequences • There has been distant metastasis at the time of diagnosis • Regular review was not performed

### Image processing

Slices with tumors or suspicious lymph nodes (cervical lymph nodes > 1 cm in length; SLN) in the axial T1-enhanced sequences of each patient were selected by a senior radiologist (with 15 years of experience) to construct the original dataset. Our experimental design contained an automatic semantic segmentation network for tumors and cervical lymph nodes and a classification neural network for tumor risk assessment. Two datasets were constructed as each network required a corresponding training dataset.

#### Dataset for semantic segmentation

Among the 500 randomly selected layers containing tumors and SLNs, tumors and SLNs were manually segmented by an otolaryngologist (with 5 years of experience) in ITK-snap software [27] and reviewed by a senior radiologist (with 15 years of experience). The slices and manually segmented regions were saved accordingly. A total of 500 slices were randomly divided into training and testing cohorts at a 4:1 ratio in the execution program.

#### Dataset for classification

**Fig. 1:**
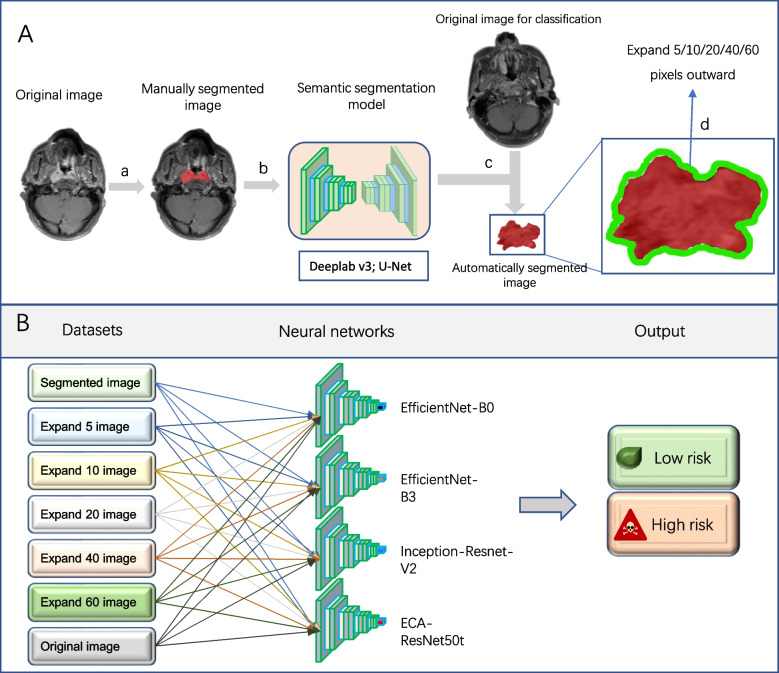
Design of the study. A: The process of building semantic segmentation model. a: Manual segmentation of tumor and suspicious lymph node regions in original images. b: Training Deeplab v3 and U-Net using manually segmented datasets. c: Automatic segmentation of datasets for classification using the trained semantic segmentation model. d. Extension by 5, 10, 20, 40, and 60 pixels outward from the segmented region to form 5 new datasets. B: The process of building classification model for predicting tumor prognosis

A total of 381 patients (2445 slices), which were divided into high-risk and low-risk groups according to PFS (patients with median PFS were classified into the high-risk group), were included in our study. Patients in each group were divided into training and test cohorts in a 4:1 ratio, and each slice was labeled consistently with the corresponding group. The original images was converted into segmented images using the trained semantic segmentation model. Segmented areas smaller than 10 × 10 pixels were automatically removed. To explore whether the inclusion of the peritumoral region helps to improve the performance of the DL model, the images were expanded to include 5, 10, 20, 40, and 60 pixels outward from the edge of the segmented region to form the corresponding expand 5 images, expand 10 images, expand 20 images, expand 40 images, and expand 60 images. Therefore, a total of seven datasets (original images, segmented images, expand 5 images, expand 10 images, expand 20 images, expand 40 images, and expand 60 images) were used for training the four neural networks for classification (Fig. 1).

### Network architecture

#### Architecture of the semantic segmentation models

Our compiling platform was based on the PyTorch library (version 1.9.0) with CUDA (version 10.0) for GPU (NVIDIA Tesla V100, nvidia corporation, Santa Clara, California, USA) acceleration on a Windows operating system (Server 2019 data center version 64 bit, 8 vCPU 32 GiB). Deeplab v3 [28] and U-Net [29] were trained by transfer learning to build the automatic segmentation models. The better-performing one was used to generate the dataset for classification. The RMSprop optimizer was used to train the models with a batch size of 32, and the initial learning rate was set to 0.001. The dropout rate of the full connected layer was set as 0.5. Both semantic segmentation models were trained for 40 epochs.

#### Architecture of the classification models

To avoid the potential impact of different preferences of different neural networks on the dataset, four common neural networks, namely Inception-Resnet-V2 [30], ECA-ResNet50t [31], EfficientNet-B3 [32], and EfficientNet-B0 [32], were trained by transfer learning to establish the classification models separately. The average performance of the four models trained on each dataset was used for evaluation. As seven datasets were used for classification, a total of 28 DL models (4*7) were established. Networks with a batch size of 32 was trained using a stochastic gradient descent optimizer with an initial learning rate of 0.001 and dropout rate of 0.5. Each model was trained for 40 epochs.

### Statistical analysis

The Dice similarity coefficient was used to evaluate the performance of the semantic segmentation models. The receiver operating characteristic curve (ROC curve) was used to evaluate the performance of each model for classification. The average area under the ROC curve (AUC), sensitivity, specificity, F1 score, and precision of the four models were used to evaluate the performance of each model on the dataset. DeLong’s test was used to compare whether the differences between the ROC curves of the models were significant. Grad-CAM images for visualizing the areas of the image that were considered by the DL models to be prognostically relevant were produced by extracting feature maps from the convolutional layers.

## Results

A total of 381 patients (2445 slices), including 194 high-risk (1394 slices) and 187 low-risk patients (1051 slices), were included in this study. Information on the age, sex, and clinical T/N stage of the included patients is shown in Table 2.

**Table 2:** Clinical characteristics of patients in the high- and low-risk cohorts

	High risk cohort	Low risk cohort
Patients	194	187
Slices	1394	1051
Age (years)		
Mean ± SD	51.22 ± 11.25	50.31 ± 10.51
< 45	48	51
45–55	87	79
> 55	59	57
Gender		
Male	139	123
Female	55	64
T stage^a^		
T1	11	24
T2	41	93
T3	82	51
T4	60	19
N stage^a^		
N0	13	37
N1	24	69
N2	123	72
N3	34	9

*SD* Standard deviation

^a^Based on the 7th edition of the American Joint Committee on Cancer (AJCC)/International Union Against Cancer staging system [16]

**Fig. 2:**
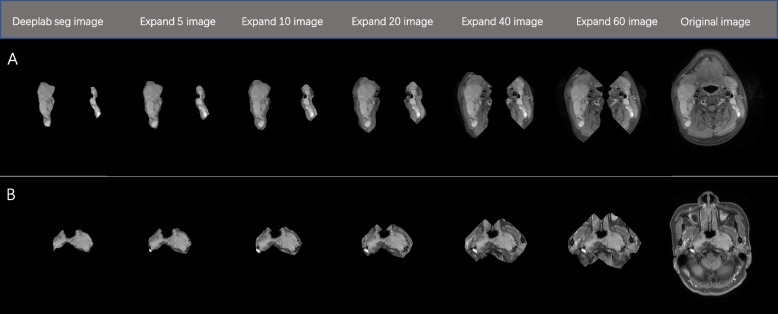
Examples of the seven datasets for training DL models. **A** and **B** represent the SLN and primary tumor, respectively, in the Deeplab seg image, expand 5 image, expand 10 image, expand 20 image, expand 40 image, expand 60 image, and original image. DL: deep learning; SLN: suspicious lymph nodes

The Dice coefficients of the two semantic segmentation models stabilized after 20 epochs. The Dice coefficients of the Deeplab v3 and U-Net after completed training were 0.741(95%CI:0.722–0.760) and 0.737(95%CI:0.720–0.754), respectively. Based on the results, Deeplab v3 was used to perform automatic segmentation of the original image and to construct a segmented image, which was named Deeplab seg image. Examples of the original image, Deeplab seg image and the constructed expand 5, 10, 20, 40, and 60 image are displayed in Fig. 2.

**Fig. 3:**
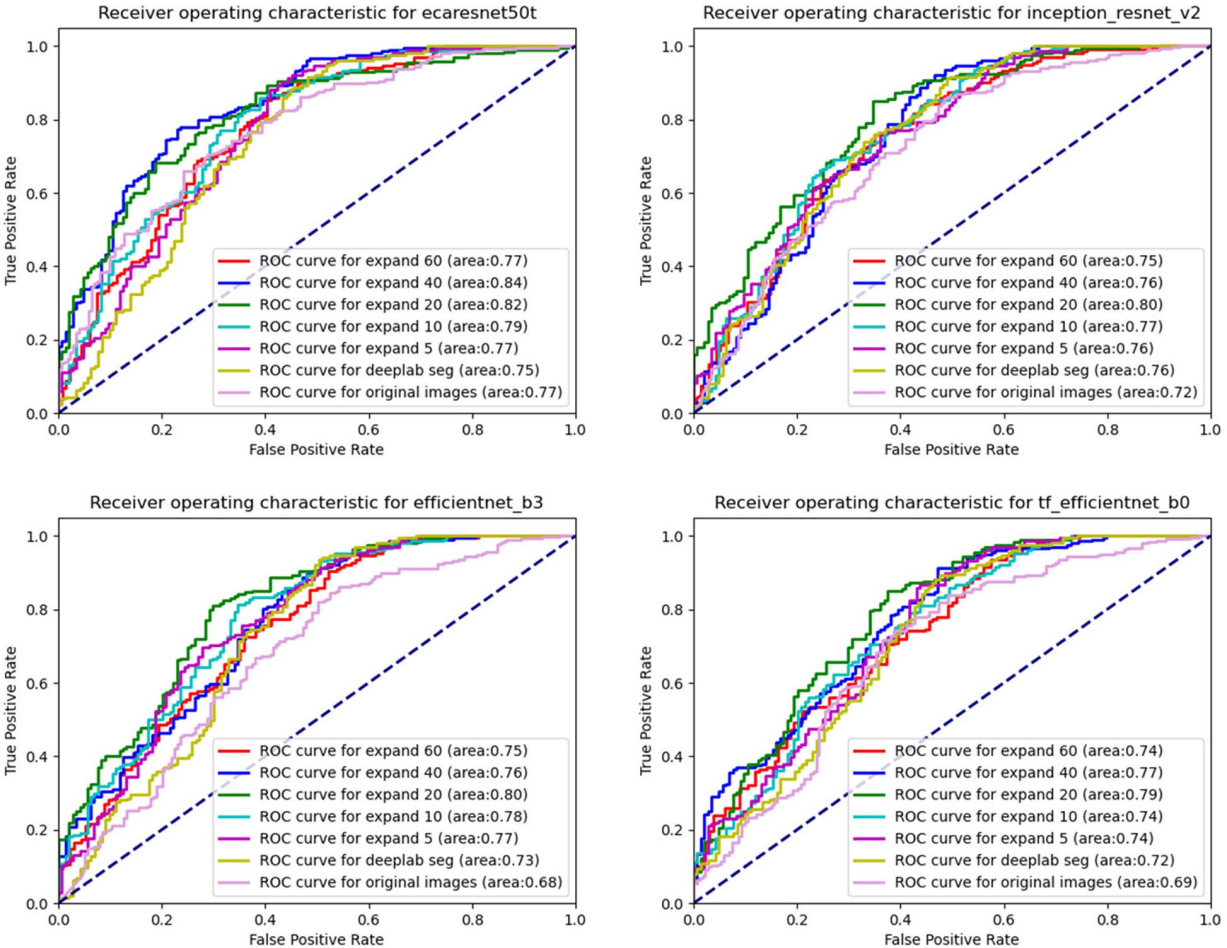
Receiver operating characteristic (ROC) curves of the ECA-ResNet50t, Inception-Resnet-V2, EfficientNet-B3, and EfficientNet-B0 trained with the seven datasets in the test cohort

**Fig. 4:**
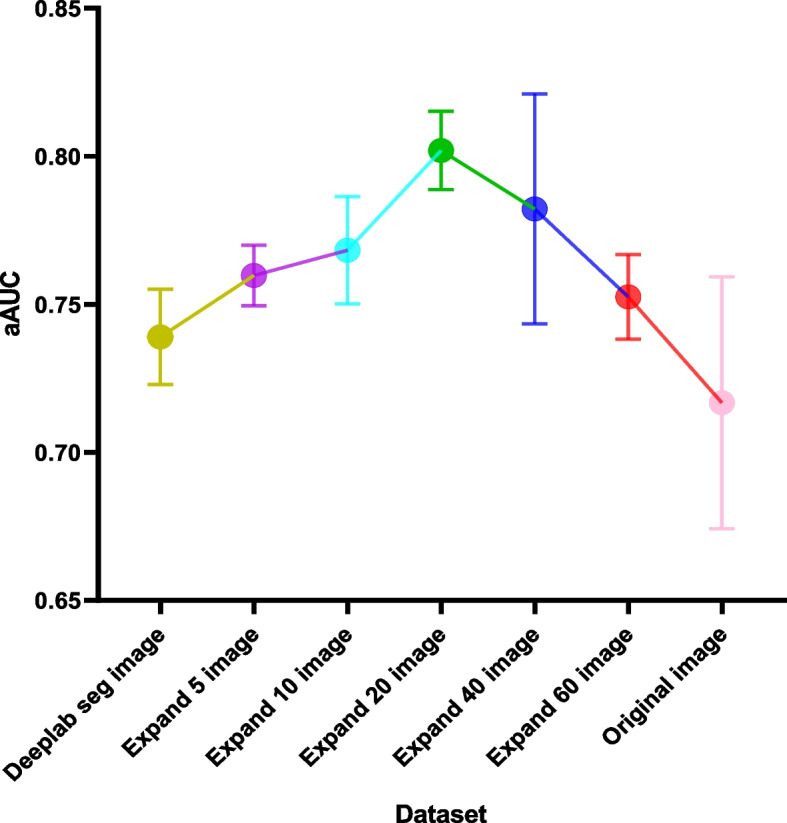
Performance of deep learning (DL) models trained with the original image, Deeplab seg image, expand 5 image, expand 10 image, expand 20 image, expand 40 image, and expand 60 image. aAUC: average area under the curve (AUC) of the four DL models based on ECA-ResNet50t, Inception-Resnet-V2, EfficientNet-B3, and EfficientNet-B0

**Fig. 5:**
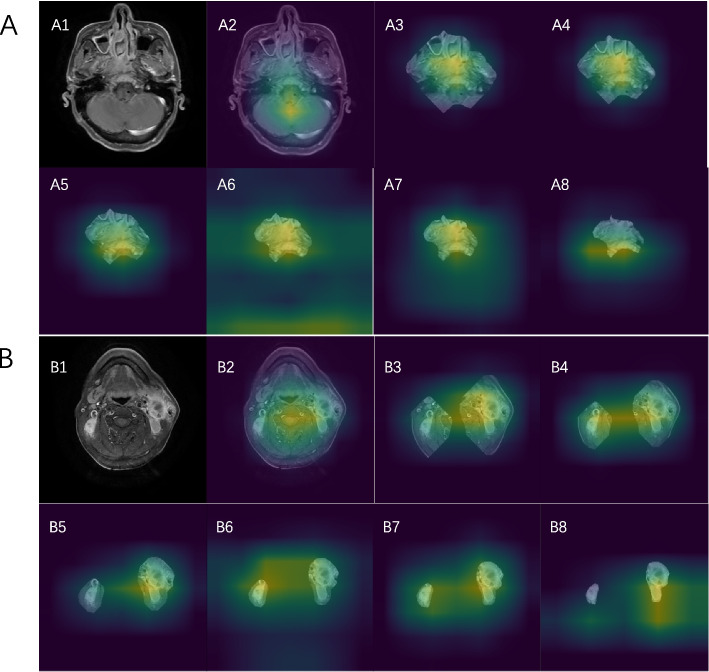
The Grad-CAM images that were generated based on EfficientNet-B0. A. A patient with nasopharyngeal carcinoma with clinical stage T4N2 was found to have tumor recurrence at 15 months after treatment (ground truth is high risk). A1 represents the original image of the patient. A2 to A8 represent Grad-CAM images generated by EfficientNet-B0 using the original image, expand 60 image, expand 40 image, expand 20 image, expand 10 image, expand 5 image, and Deeplab seg image, respectively. B. A patient with nasopharyngeal carcinoma with clinical stage T2N3 and no tumor recurrence at 43 months of follow-up (ground truth is low risk). B1 represents the original image of the patient. B2 to B8 represent Grad-CAM images generated by EfficientNet-B0 using the original image, expand 60 image, expand 40 image, expand 20 image, expand 10 image, expand 5 image, and Deeplab seg image, respectively. The yellow bright area indicates the region considered by the model to be most relevant to the prognosis of the tumor, followed by the green color. As it can be seen in the A2 and B2 plots that the DL model based on the original image classifies high- and low-risk patients on the basis of features that appear to be unreasonable in the physician’s experience as the bright yellow areas are concentrated around the brainstem. This situation occurs in a very large number of cases

The AUC, sensitivity, specificity, precision, and f1 scores of the seven DL models based on ECA-ResNet50t, Inception-Resnet-V2, EfficientNet-B3, and EfficientNet-B3 trained on the original image, Deeplab seg image, extended 5 image, extended 10 image, extended 20 image, extended 40 image, and extended 60 image are shown in Table 3 and Fig. 3. The AUC of the DL model based on ECA-ResNet50t trained on the extended 20 images was significantly higher than that of the DL model trained on the original image and Deeplab seg image, with *p*-values of 0.013 and 0.032, respectively. There was no significant difference between the performance of the DL model trained on the original image and Deeplab seg image (*p* = 0.203). The AUC of the DL model based on Inception-Resnet-V2 trained on the extended 20 images was also significantly higher than that of the DL model trained on the original image (*p* = 0.010), while there was no significant difference between the performance of the DL model trained on the extended 20 images and Deeplab seg image (*p* = 0.056). The AUC of the DL model based on EfficientNet-B3 trained on the extended 20 images was significantly higher than that of the DL model trained on the original image and Deeplab seg image, with *p*-values of 0.007 and 0.012, respectively. The performance of the DL model trained on the Deeplab seg image was significantly higher than that of the DL model trained on the original image (*p* = 0.041). The AUC of the DL model based on EfficientNet-B0 trained on the extended 20 images was significantly higher than that of the DL model trained on the original image and Deeplab seg image, with p-values of 0.009 and 0.034, respectively, while there was no significant difference between the performance of the DL model trained on the original image and Deeplab seg image (*p* = 0.122). The average AUC (aAUC) of the four deep neural networks trained with the original image, Deeplab seg image, expand 5 image, expand 10 image, expand 20 image, expand 40 image, and expand 60 image were 0.717 ± 0.043, 0.739 ± 0.016, 0.760 ± 0.010, 0.768 ± 0.018, 0.802 ± 0.013, 0.782 ± 0.039, and 0.753 ± 0.014, respectively (Fig. 4). The DL models trained with the expand 20 images obtained the highest aAUC (0.802 ± 0.013), whereas the models trained with the original image (0.717 ± 0.043) and Deeplab seg image (0.739 ± 0.016) performed the worst. The Grad-CAM images generated based on EfficientNet-B0 is used as an example to show the predictive basis of the model (Fig. 5).

**Table 3:** Performance of seven DL models based on ECA-ResNet50t, Inception-Resnet-V2, EfficientNet-B3, and EfficientNet-B3 trained on the original image, Deeplab seg image, extended 5 image, extended 10 image, extended 20 image, extended 40 image, and extended 60 image

Dataset	Neural network	Sensitivity	Specificity	F1 score	Precision	AUC	95%CI	aAUC (SD)
Original image	ECA-ResNet50t	0.810	0.724	0.803	0.796	0.774	0.730–0.818	0.717 (0.043)
	Inception-Resnet-V2	0.738	0.695	0.750	0.763	0.722	0.685–0.759	
	EfficientNet-B3	0.656	0.695	0.696	0.741	0.676	0.635–0.721	
	EfficientNet-B0	0.659	0.743	0.712	0.773	0.695	0.644–0.746	
Deeplab seg image	ECA-ResNet50t	0.781	0.695	0.777	0.773	0.745	0.704–0.786	0.739 (0.016)
	Inception-Resnet-V2	0.774	0.729	0.783	0.791	0.759	0.719–0.798	
	EfficientNet-B3	0.706	0.752	0.746	0.791	0.727	0.684–0.770	
	EfficientNet-B0	0.688	0.771	0.740	0.800	0.725	0.678–0.773	
Expand 5 image	ECA-ResNet50t	0.803	0.719	0.797	0.792	0.768	0.728–0.809	0.760(0.010)
	Inception-Resnet-V2	0.778	0.733	0.786	0.795	0.761	0.718–0.804	
	EfficientNet-B3	0.746	0.790	0.783	0.825	0.765	0.718–0.812	
	EfficientNet-B0	0.710	0.790	0.760	0.818	0.745	0.703–0.787	
Expand 10 image	ECA-ResNet50t	0.821	0.738	0.813	0.806	0.786	0.739–0.832	0.768 (0.018)
	Inception-Resnet-V2	0.789	0.738	0.794	0.800	0.766	0.730–0.802	
	EfficientNet-B3	0.756	0.800	0.793	0.834	0.777	0.732–0.822	
	EfficientNet-B0	0.706	0.790	0.758	0.817	0.744	0.707–0.781	
Expand 20 image	ECA-ResNet50t	0.849	0.767	0.839	0.829	0.817	0.766–0.868	0.802 (0.013)
	Inception-Resnet-V2	0.817	0.776	0.823	0.829	0.801	0.757–0.845	
	EfficientNet-B3	0.789	0.829	0.822	0.859	0.805	0.754–0.856	
	EfficientNet-B0	0.749	0.833	0.799	0.857	0.785	0.732–0.837	
Expand 40 image	ECA-ResNet50t	0.875	0.790	0.861	0.847	0.84	0.783–0.897	0.782 (0.039)
	Inception-Resnet-V2	0.774	0.738	0.785	0.797	0.758	0.713–0.803	
	EfficientNet-B3	0.742	0.786	0.780	0.821	0.761	0.721–0.802	
	EfficientNet-B0	0.735	0.819	0.785	0.844	0.77	0.725–0.815	
Expand 60 image	ECA-ResNet50t	0.806	0.724	0.801	0.795	0.773	0.732–0.814	0.753 (0.014)
	Inception-Resnet-V2	0.767	0.724	0.777	0.787	0.75	0.709–0.791	
	EfficientNet-B3	0.728	0.771	0.766	0.809	0.747	0.708–0.786	
	EfficientNet-B0	0.703	0.786	0.754	0.813	0.74	0.699–0.781	

*AUC* Area under the curve, *aAUC* Average area under the curve (AUC) of the four DL models based on ECA-ResNet50t, Inception-Resnet-V2, EfficientNet-B3, and EfficientNet-B0, *SD* Standard deviation

## Discussion

In this study, four common neural networks were trained with the original, segmented, and the five new constructed datasets to establish DL models for the prognosis prediction of NPC. The results show that the DL models trained with the segmented region and the original images performed the worst (aAUC was 0.739 ± 0.016 and 0.717 ± 0.043 respectively). The performance of the models gradually improved when the segmented area was gradually extended outward by 5, 10, and 20 pixels, and reached its maximum when it was extended by 20 pixels (aAUC was 0.802 ± 0.013). Then, the performance of the model gradually decreased when the area was extended beyond 20 pixels. This confirms the predictive value of peritumor area images for the prognosis of nasopharyngeal carcinoma.

A review titled “Nasopharyngeal carcinoma” published in The Lancet in 2019 proposed 18 research questions on NPC that remain to be answered, and two of them were about AI and NPC: “How can reliable radiomics models for improving decision support in NPC be developed?” and “How can artificial intelligence automation for NPC treatment decisions be applied?” [33]. These two issues remain relevant today, as DL models are still facing the challenges of low reliability and practicality. Risk assessment of NPC is a focal topic in this field. Traditionally, TNM staging of tumors has been used to assess tumor risk, where T is the relevant information about the primary tumor. According to the 7th and 8th edition of the American Joint Committee on Cancer/International Union Against Cancer staging systems, the T-staging of NPC is determined based on the relationship between the tumor and the surrounding anatomical structure and does not contain information from inside the tumor [24, 25]. According to the literature, T-staging has good predictive performance for the prognosis of NPC [9, 19, 26]. However, in most studies, only the tumor region was delineated as the region of interest in the radiomic approach, and peritumor information was obliterated. This may cause a partial loss of information related to prognosis, which leads to a limited performance of the model. Simultaneously, there are several studies using DL to establish a prognostic prediction model for NPC in which the original image was incorporated for model training [4–7]. However, original images contain a large amount of noise, such as uninvaded cerebellar, nasal, and temporal regions far from the tumor, which may provide only very limited prognostic information, and most of the pixels in these regions are noise. The inclusion of these regions may lead to a decrease in model performance and increase the requirement for the amount of training data. As a result, the models developed in these studies may not have fully exploited the prognostic information embedded in the images for NPC. By analogy, when predicting the prognosis of other tumors, such as gastric [34], breast [35], and cervical cancer [36], based on radiomics or CNN techniques, the inclusion of peritumor image may be able to improve the performance of the prediction model.

**Fig. 6:**
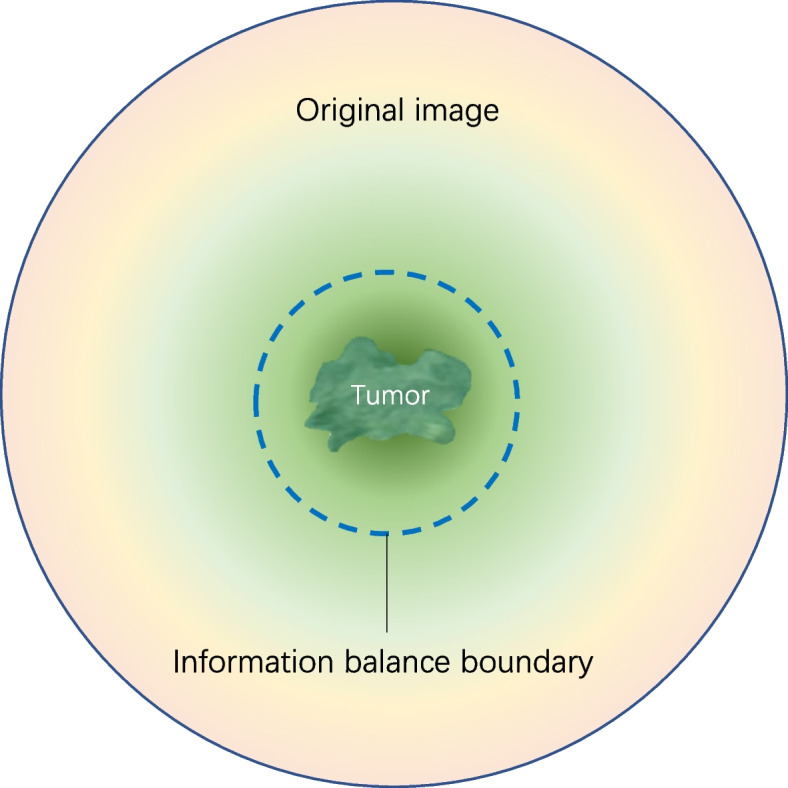
Information balance boundary visualization of the information content of the regions

Before the tumor invades the surrounding anatomical structures, some patients have developed cellular-level tumor microfiltration which does not result in significant changes in image signal and is difficult to correlate with patient prognosis with the naked eye. However, this may indeed be indicative of tumor progression and may therefore be closely related to the patient’s prognosis. Neural networks that analyze images at the pixel level can capture these infiltrative features, which are difficult to distinguish with the naked eye, and correlate them with the patient’s prognosis. Therefore, we logically assume that the prognostic information decreases progressively outward from the tumor region, while the noise increases progressively, and there should be an information balance boundary. Better performance could be achieved by incorporating the region within this information balance boundary to train the prognostic prediction model (Fig. 6).

In this study, to avoid the influence of the neural network structure on the results, four common neural networks were selected, and aAUCs were calculated to evaluate the impact of using different types of images. The results show that the models trained with the segmented region and the original images performed the worst. The performance of the models gradually improved when the segmented area was gradually extended outward by 5, 10, and 20 pixels, and reached its maximum when it was extended by 20 pixels. Then, the performance of the model gradually decreased when the area was extended beyond 20 pixels. This result can be explained by the hypothesis of the information balance boundary mentioned above: although the noise in the original image has been removed to the maximum extent in the Deeplab seg image, the peritumoral region, which contains considerable prognostic information about the tumor, has also been eliminated. Inadequate inclusion of prognostic information leads to unsatisfactory performance of the model. In the process of outward expansion, the prognostic information was gradually incorporated sufficiently so that the model performance gradually improved. However, more noise and less prognostic information are included as the region is extended further, which causes the performance of the model to begin to degrade, with the worst performance when incorporating the full image. The information balance boundary of our dataset was determined to be 20 pixels outward from the tumor. However, different datasets may have different information balance boundaries, which need to be confirmed by including more multicenter data.

The cost of collecting information related to tumor prognosis is extremely high, especially for tumors such as NPC, which have an overall 5-year survival rate of 80% [37], as it involves a considerable period of review and follow-up. Therefore, it is extremely important to train models that perform well with limited data. Computer experts approach this problem from the perspective of mathematical algorithms. However, each medical task combined with AI includes a special medical background knowledge. How to leverage the specific medical knowledge behind these tasks and provide more adequate information to make the trained AI models perform better is one of the main tasks/contributions of physicians in this field.

This study has some limitations. First, the number of cases included in this study was relatively small compared with that in other studies, which may have affected the performance of the models. Considering that this study was not designed to establish an excellent prognostic prediction model for NPC but was a methodological study, the data requirements were not demanding. Second, this study was a single-center study, and it is still unclear whether there are different information balance boundaries for the prognosis of NPC in multicenter data.

## Conclusion

The peritumoral region on the MRI image of NPC contains information related to prognosis, and the inclusion of this information could improve the performance of the model for predicting the prognosis of the tumor.

## Data Availability

The data and material are available through the corresponding author.

## Abbreviations

AI: Artificial intelligence
AUC: Area under the curve
DL: Deep learning
NPC: Nasopharyngeal carcinoma
PFS: Progression-free survival
ROC: Receiver operating characteristic
SLN: Suspicious lymph nodes
